# Preclinical development of 1B7/CD3, a novel anti-TSLPR bispecific antibody that targets *CRLF2*-rearranged Ph-like B-ALL

**DOI:** 10.1038/s41375-023-02010-y

**Published:** 2023-08-26

**Authors:** Ze Tian, Chunhua Shi, Guojun Yang, Jason K. Allen, Qing Shi, Amin AL-Shami, Jill Wardell Olson, Melinda G. Smith, Qing Chang, Jasbir Kaur, Junping You, Timothy E. Lofton, Michelle A. Gonzalez, Qi Zhang, DongXing Zha, Sarah K. Tasian, Nitin Jain, Marina Y. Konopleva, Timothy Heffernan, Jeffrey J. Molldrem

**Affiliations:** 1 ORBIT Platform, The University of Texas MD Anderson Cancer Center, Houston, TX USA; 2 Department of Leukemia, Division of Cancer Medicine, The University of Texas MD Anderson Cancer Center, Houston, TX USA; 3 Division of Oncology and Center for Childhood Cancer Research, Children’s Hospital of Philadelphia; Department of Pediatrics and Abramson Cancer Center, University of Pennsylvania School of Medicine, Philadelphia, PA USA; 4 Translational Research to Advance Therapeutics and Innovation in Oncology (TRACTION), The University of Texas MD Anderson Cancer Center, Houston, TX USA; 5 Department of Hematopoietic Biology & Malignancy, Division of Cancer Medicine, The University of Texas MD Anderson Cancer Center, Houston, TX USA

**Keywords:** Cancer immunotherapy, Immunotherapy

## Abstract

Patients harboring *CRLF2*-rearranged B-lineage acute lymphocytic leukemia (B-ALL) face a 5-year survival rate as low as 20%. While significant gains have been made to position targeted therapies for B-ALL treatment, continued efforts are needed to develop therapeutic options with improved duration of response. Here, first we have demonstrated that patients with *CRLF2*-rearranged Ph-like ALL harbor elevated thymic stromal lymphopoietin receptor (TSLPR) expression, which is comparable with CD19. Then we present and evaluate the anti-tumor characteristics of 1B7/CD3, a novel CD3-redirecting bispecific antibody (BsAb) that co-targets TSLPR. In vitro, 1B7/CD3 exhibits optimal binding to both human and cynomolgus CD3 and TSLPR. Further, 1B7/CD3 was shown to induce potent T cell activation and tumor lytic activity in both cell lines and primary B-ALL patient samples. Using humanized cell- or patient-derived xenograft models, 1B7/CD3 treatment was shown to trigger dose-dependent tumor remission or growth inhibition across donors as well as induce T cell activation and expansion. Pharmacokinetic studies in murine models revealed 1B7/CD3 to exhibit a prolonged half-life. Finally, toxicology studies using cynomolgus monkeys found that the maximum tolerated dose of 1B7/CD3 was ≤1 mg/kg. Overall, our preclinical data provide the framework for the clinical evaluation of 1B7/CD3 in patients with *CRLF2*-rearranged B-ALL.

## Introduction

Acute lymphocytic leukemia (ALL), the most common form of leukemia in childhood and adolescence, is characterized by clonal expansion of lymphoid progenitor cells present in the bone marrow (BM), blood, and extramedullary sites [1]. In the past decade, tremendous progress has been made in the treatment of ALL with the development of targeted therapies, including tyrosine kinase inhibitors of BCR::ABL1, monoclonal antibodies, bispecific antibodies (BsAb), and chimeric antigen receptor T- cell therapy targeting cell surface antigens [2]. However, while survival of childhood ALL approaches 90%, only 30–40% of adult patients achieve long-term remission [3, 4].

BCR-ABL1-like, or Ph-like, ALL is a recently identified category of B-ALL with a poor prognosis [5, 6]. This high-risk disease carries a gene expression signature similar to that of Ph+ ALL but without the BCR::ABL1 translocation, as well as genomic alterations that activate several types of kinase signaling pathways [7, 8]. Rearrangement of *cytokine receptor‐like factor 2* (*CRLF2*), which encodes TSLPR [9], is found in approximately 50% of patients diagnosed with Ph-like B-ALL and is common in patients with Down syndrome [10]. *CRLF2* rearrangements occur either as a translocation to the immunoglobulin heavy-chain enhancer region (*IGH::CRLF2*) or by a deletion of upstream PAR1 that leads to the fusion of *CRLF2* to adjacent *P2RY8* [11]. Activating point mutations of Phe232Cys (F232C) have also been detected [8, 12, 13]. Subsequently, genetically aberrant *CRLF2* dysregulates TSLPR expression and cooperates with mutations in JAK kinases to activate the JAK/STAT pathway, signaling downstream of the TSLPR/IL-7α heterodimeric receptor complex [14]. Importantly, TSLPR-dependent signaling promotes B-cell leukemogenesis thus suggesting that targeting TSLPR with blocking or depleting strategies could be therapeutically promising. Accordingly, administration of the TSLPRα blocking antibody 1E10 has been shown to inhibit both TSLP-triggered cell proliferation and STAT transcription factor activation [15], and TSLPR chimeric antigen receptor T-cell (CAR-T) therapy has been shown to eradicate human CRLF2-overexpressing ALL in xenograft models [16].

In recent years, redirection of T cells against tumors using BsAb such as blinatumomab, a bispecific monoclonal antibody that targets CD19, has been shown to induce high response rates in relapsed/refractory B-ALL patients. One major advantage of BsAb over CAR-T is its availability “off the shelf”, which reduces cost and eschews the time needed for CAR-T production [17]. However, responses with blinatumomab are relatively short, with a 12-month event-free survival of approximately 20% [18, 19]. Further, clinical use of blinatumomab is limited due to a short half-life and frequent antigen loss or downregulation [20, 21]. Here, we report on a novel anti-TSLPR BsAb, named 1B7/CD3. We characterized the biophysical properties, in vitro function, as well as demonstrated the efficacy, safety, and prolonged half-life. Together, our results suggest 1B7/CD3 be a promising novel treatment for patients with *CRLF2*-rearranged B-ALL.

## Methods

### Antibody discovery, top clinical lead selection, and BsAb construction

H2L2 human transgenic mice were purchased from Harbor BioMed (Boston, MA). Single B-cell Cloning (SBCC) technology was utilized to amplify variable heavy and variable light gene regions from the cDNA of TSLPR-Fc immunized H2L2 mouse memory B-cells. The naturally paired variable regions were cloned into pcDNA3.1 + hIgG1 and hKappa expression vectors for subsequent ExpiCHO high throughput transient expression in 96-well format. Following Protein-A magnet beads purification, binding avidity was assayed by Bio-Layer Interferometry (BLI) with anti-Human IgG Fc capture (AHC) biosensors. Positive clones were then expressed for further cell surface EC50, cross-reactivity, epitope binning, ligand blockage, and stability assays.

To generate the 1B7/CD3 BsAb, 1B7 heavy and light chain gene regions were cloned into hIgG1 or hKappa pcDNA3.1+ expression vectors respectively. Xencor CD3 antibody and heterodimeric Fc technology were applied for the BsAb. 1B7/CD3 was expressed in ExpiCHO cells through transient transfection and then purified using Protein-A and cation affinity columns (AKTA FPLC system). The binding of 1B7/CD3 with the TSLPR/CD3 antigen was also tested by BLI.

### Cell lines and primary B-ALL samples

B-ALL cell lines MHH-CALL4 (DSMZ, Germany) and REH (ATCC, Manassas, VA) were used in the study. REH-TSLPR-Luc [16] was created by sequential lentiviral transduction with a human CRLF2 plasmid (GeneCopoeia, Rockville, MD) and a firefly luciferase plasmid (Addgene, Watertown, MA) followed by FACS sorting. Primary B-ALL cells were from MD Anderson Cancer Center (MDACC) with informed consent obtained, and BOS-1 patient-derived xenograft (PDX) models harboring the *IGH::CRLF2* rearrangement were obtained from Dr. David Weinstock [21] at Dana-Farber Cancer Institute. Peripheral blood mononuclear cells (PBMC) from human volunteers were isolated from Buffy Coats (Gulf Coast Blood Bank, Houston, TX) using a Ficoll-Paque density gradient (GE Healthcare, Chicago, IL), and the protocol was approved by insitiutional review board. Human bone marrow was purchased from Lonza (Basel, Switzerland). Cynomolgus bone marrow was purchased from Humancells Bioscience (Milpitas, CA).

### TSLPR expression and functional assays

Primary B-ALL samples were stained with anti-CD45, anti-CD19, and anti-TSLPR (BioLegend, San Diego, CA). For functional assays, GFP + REH-TSLPR cells were incubated with 1B7/CD3 or a control BsAb before addition of CD8 + T cells. The levels of the activation marker CD69 were shown on CD3 + CD8 + GFP- cells. All sample acquisition was performed using fluorescence-activated cell sorting (FACS) and analyzed using FlowJo v10.5 (Ashland, OR). For killing assay, REH-TSLPR, MHH-CALL4, and Alexa Fluor 750 tagged T cells or bone marrow cells were incubated with different concentrations of 1B7/CD3 or a control BsAb before adding T cells. Cell viability was determined by FACS.

### Animal models

#### Animal, dosing, and tumor measurement

All animal experiments were conformed to the relevant regulatory standards and approved by the Institutional Animal Care and Use Committee at MDACC. Sample size selection was based on literature [22]. NOD.CgPrkdc^scid^ Il2rg^tm1Wjl^/SzJ (NSG) mice (The Jackson Laboratory) were intravenously (iv) injected with Reh-TSLPR-Luc cells or Bos-1 PDX cells. Once bioluminescence from REH reached around 10^7^ p/sec or BOS-1 number reached around 20% in blood, 10 × 10^6^ PBMC/mouse were injected I.V. for humanization, followed by stratified randomization with 5 mice in each group and treatment with 1B7/CD3 or vehicle via intraperitoneal (I.P.) injection weekly for three weeks. Leukemia burden was assessed by imaging or FACS.

#### T cell dynamic, activation, and phenotyping

Blood was collected from mice once weekly for dynamic T cell profiling. Briefly, blood was processed to single cell suspension and stained with BV510 Ghost dye (Tonbo Biosciences), anti-mouse CD45 (Invitrogen), anti-human CD45, CD3, CD19 (BD Horizon), TSLPR, CD4, CD8, CD69, CD62L CD45RO, and CD45RA (Biolegend). T cell state was determined by phenotypic markers and data were acquired on FACS and analyzed using FlowJo v10.5.

#### Histology

For histological analysis, formalin-fixed mouse tissues were embedded in paraffin, sectioned, stained with hematoxylin and eosin (H&E), CD3 (Serotec), or HLA-A (Abcam) for immunohistochemistry (IHC) and imaged by a Pannoramic 250 scanner.

### Pharmacokinetic (PK) analysis of 1B7/CD3 in mice

Single dose PK was studied in naive and PBMC humanized NSG mice. For PK analysis in NSG mice, animals were randomized into treatment groups and were administered 0.3, or 1 mg/kg 1B7/CD3 by an I.P. injection. For PK analysis in humanized NSG mice, 1 × 10^7^ PBMC was administered through iv injection into mice 10 days prior to randomization for dosing. Blood samples were collected at indicated time points and processed to plasma for bioanalytical and PK analyses.

### Toxicity of 1B7/CD3 in cynomolgus monkeys

#### General toxicity

In the exploratory dosing finding toxicity study, three adult, female cynomolgus monkeys were randomized into treatment groups and received 0.3, 1, or 3 mg/kg 1B7/CD3 via iv bolus on Day 1 and Day 8. Monkeys were monitored closely for moribundity and mortality. Whole blood was processed for clinical pathology at indicated time points. Organs/tissues were harvested for gross finding and histopathology (Supplemental Table 3).

1B7/CD3 plasma exposure, 1B7/CD3-induced T cell activation and cytokine release.

Blood was collected at indicated time points to evaluate 1B7/CD3 exposure, T cell activation, and cytokine release. Briefly, blood was processed into plasma for bioanalytical analysis. Concentrations of 1B7/CD3 in plasma were measured as described in PK assay. Cytokine release was analyzed with a Multiplex Non-Human Primate Cytokine Magnetic bead-based immunoassay (Millipore) using Luminex technology. For T cell activation, blood cells were stained with anti-CD3, CD69, and CD25 antibodies (BioLegend). Data were acquired by FACS and analyzed using FlowJo v10.5.

Source of all antibodies is listed in Supplementary Table 4.

#### Statistical analysis

The investigators were not blinded to the group allocation during the experiment. Statistical drug-treated and control groups were determined by using a student’s *t*-test or one-way analysis of variance (ANOVA) using GraphPad Prism 9. Nonparametric tests were used when *t*-test were not applicable. Significant differences were indicated by *p* < 0.05.

### Data sharing statement

For original data, please contact ztian1@mdanderson.org.

## Results

### Identification of anti-TSLPR antibodies and 1B7/CD3 bispecific construction

**Fig. 1:**
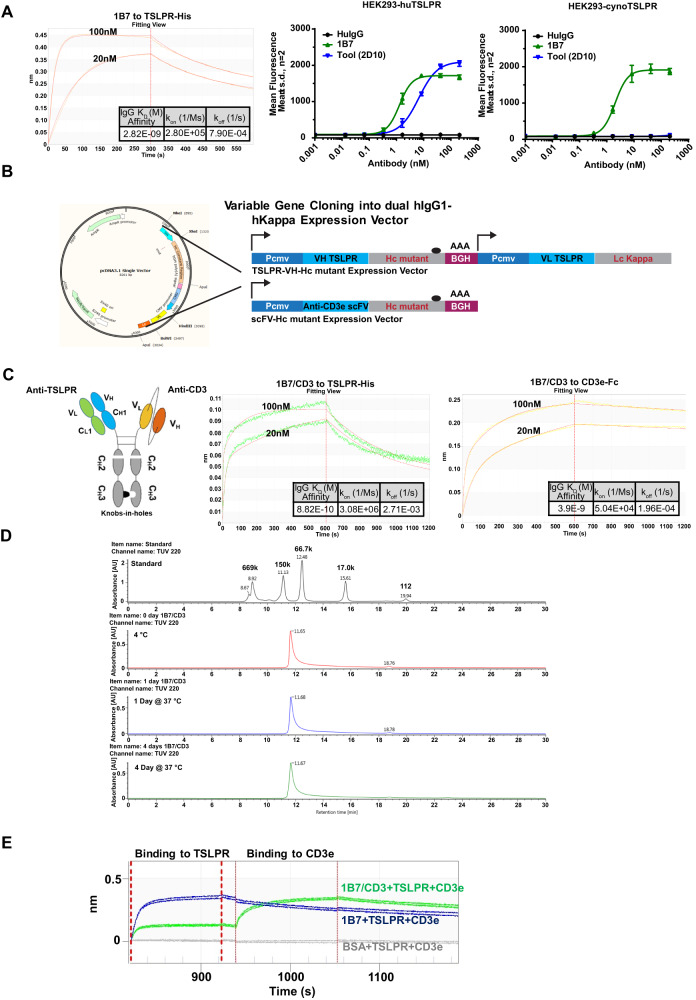
Development and characteristics of the 1B7/CD3 bispecific antibody (BsAb). **A** Octet and cell surface binding of the top clone 1B7 with TSLPR-His (lab prepared monomer structure confirmed), human TSLPR (huTSLPR, and cynomolgus monkey TSLPR (cynoTSLPR) (**B**) Design of both TSLPR-VH-Hc mutant and anti-CD3e-VH-Hc mutant (scFv) vectors in pcDNA3.1 for bispecific monoclonal antibody expression. **C** Left, cartoon of the 1B7/CD3 BsAb with a non-functional Fc region; Middle and right panel, 1B7/CD3 BsAb was tested for binding to either antigen TSLPR or CD3e. **D** The stability of 1B7/CD3 BsAb was tested by ultra-high performance liquid chromatography-size exclusion chromatography (UHPLC-SEC) at indicated time points and temperatures. **E** Tandem binding of 1B7/CD3 BsAb to both TSLPR and CD3e antigens was assayed by Bio-Layer Interferometry. 1B7 alone served as the positive control for TSLPR binding, and negative control for CD3e binding. A BSA loaded sensor with TSLPR and CD3e served as the negative control for both antigen binding.

To identify anti-TSLPR antibodies, fully humanized TSLPR-specific monoclonal antibodies were first generated from immunized H2L2 Harbour mice using SBCC technology (Supplementary Fig. 1A–C), and a total of seventy clones with unique sequences were discovered with variable affinities (Supplementary Fig. 1D–H). Clone 1B7, with an affinity of 2.82 nM (Fig. 1A), was selected as the final candidate based on cynomolgus cross-reactivity (Fig. 1A), and developmental properties.

To generate bispecific 1B7/CD3 constructs, the 1B7 heavy and light chains were cloned into hIgG1 or hKappa expression vectors, respectively (Fig. 1B). Xencor’s CD3 BsAb technology [23] was applied to create a BsAb with an Fc-silent region and an affinity of 0.88 nM to TSLPR (Fig. 1C). The BsAb was found to be stable without any apparent aggregation or degradation (Fig. 1D). Tandem binding of CD3 and TSLPR by the BsAb was then confirmed by BLI (Fig. 1E).

### 1B7/CD3 activates T cells and triggers antigen-specific tumor lysis

**Fig. 2:**
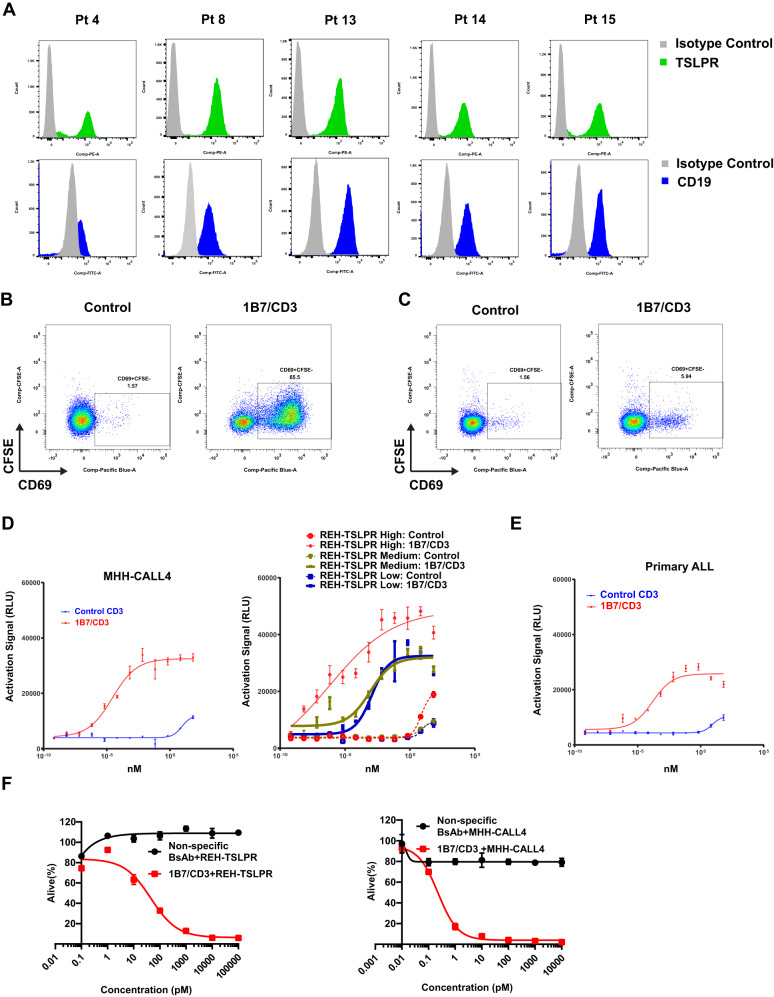
1B7/CD3 bispecific antibody (BsAb) induced a strong antigen-specific T cell activation and tumor cell killing in vitro. **A** Comparable expression of TSLPR and CD19 in primary B-cell acute lymphocytic leukemia (B-ALL) samples. TSLPR and CD19 expression were determined on gated live CD45/TSLPR or CD45/CD19 double-positive cells respectively. **B** Activation of CD8 + T cells by 1B7/CD3 BsAb. REH-TSLPR cells (GFP+) were incubated with 1B7/CD3 BsAb or a control (inactive) BsAb before addition of human primary CD8 + T cells followed by culturing for 48 h. Levels of the activation marker CD69 are shown on CD3 + CD8 + GFP− cells. **C** 1B7/CD3 BsAb shows low levels of activation with human PBMC. 1B7/CD3 or inactive control BsAb 10 µg/ml were added to peripheral blood mononuclear cells (PBMC) from a normal donor. Levels of CD69 expression, as an indication of CD8 + T cell activation, were measured. **D**, **E** Expression of the NFAT luciferase reporter was triggered by 1B7/CD3 BsAb, but not the control antibody, in a reporter assay. **D** Low, medium, and high TSLPR expression (~1000, 2500, and 30,000 receptors/cell, respectively) in REH cells. Expression of TSLPR in (**D**) MHH-CALL4 ALL cells (native expressers of TSLPR, ~4000 copies) or in (**E**) cells from a primary patient sample (~6000 copies/cell). Cells were coated with 1B7/CD3 BsAb or control antibody before the addition of Jurkat cells expressing the NFAT luciferase reporter. The NFAT expression signal was measured 24 h. post activation and was expressed in relative light unit (RLU). **F** REH-TSLPR and MHH-CALL4 ALL cells were incubated with different concentrations of 1B7/CD3 BsAb or control antibody before adding human T cells at a 5:1 ratio (E: T). Flow cytometry was used to determine the cell viability after 48 h. treatment.

We first characterized antigen levels by assessing TSLPR expression in primary Ph-like B-ALL patient specimens harboring *CRLF2* rearrangements. Our data revealed comparably high expression of TSLPR and CD19 across patients (Fig. 2A), which strongly supported targeting TSLPR as a novel approach for B-ALL therapy.

The ability of 1B7/CD3 to activate T cells and to kill B-ALL cells in vitro was then investigated. Activation of CD8 T cells, as demonstrated by the strong upregulation of CD69 when exposed to REH-TSLPR cells (Fig. 2B), was induced by 1B7/CD3 but not by the control antibody, thus indicating that CD8 T cell activation was antigen specific. Interestingly, in a similar experiment that used normal PBMC as the target, 1B7/CD3 only induced minimal activation of CD8 T cells, suggesting that TSLPR levels in normal PBMC were too low to trigger a strong T cell response (Fig. 2C). Additionally, incubation with 1B7/CD3 was shown to induce the activation of Jurkat T cells equipped with an NFAT luciferase reporter when co-cultured with REH-TSLPR cells expressed escalating levels of TSLPR, MHH-CALL4 ALL cells, and primary ALL blasts (Fig. 2D–E). Furthermore, 1B7/CD3 treatment was selectively efficacious at mediating the killing of REH-TSLPR and MHH-CALL4 cells, but not normal T cells or BM cells (Fig. 2F, Supplementary Fig. 2A–C). Overall, our data suggest that 1B7/CD3 induced antigen-specific activation of T cells leading to the elimination of ALL cells in vitro.

### 1B7/CD3 inhibits tumor growth in xenograft models

**Fig. 3:**
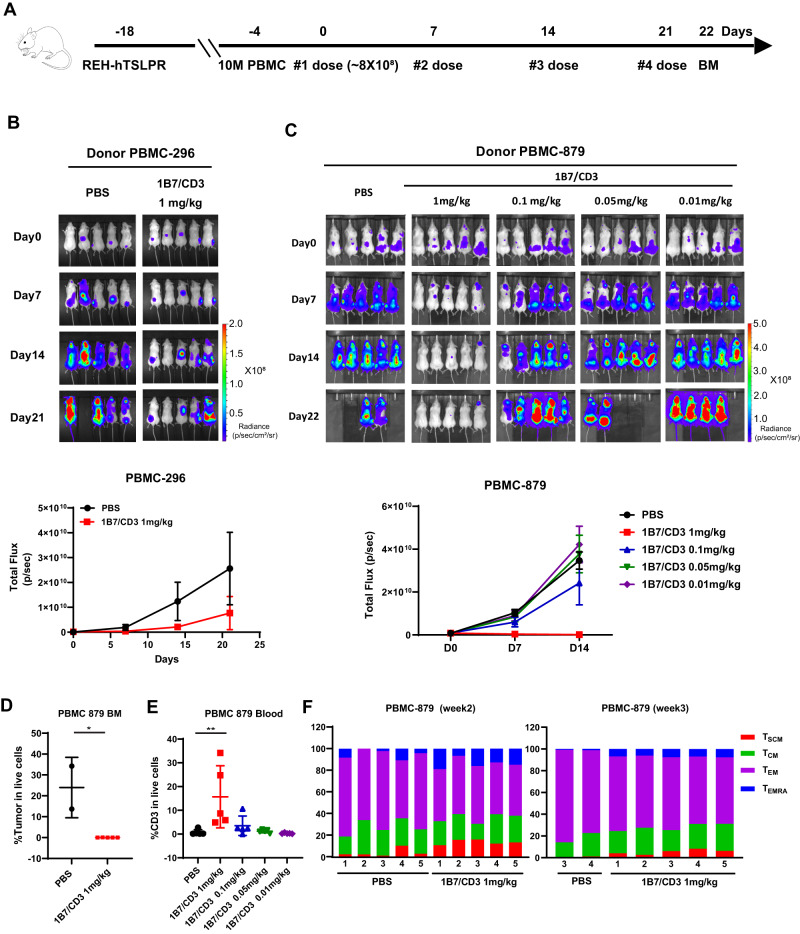
In vivo activity of 1B7/CD3 bispecific antibody (BsAb) in the REH-TSLPR cell line derived xenograft model. **A** Schematic of experimental design. Female NSG mice at 6–10 weeks old received a tail vein injection of 0.1 M REH-TSLPR human acute lymphoblastic leukemia (ALL) cells per mouse. Two weeks after tumor inoculation, 10 M PBMC/mouse were intravenously injected for humanization. Three days after the PBMC injection, mice were randomized into control and treatment groups. Mice were either treated with vehicle control or 1B7/CD3 at different doses at indicated time points. Tumor burden was assessed by imaging using IVIS (PerkinElmer). **B** Imaging (top) and radiance plot (bottom) monitoring tumor growth in donor 296 humanized mice (Donor PBMC-296) treated with control (PBS) or 1B7/CD3 (1 mg/kg) at indicated time points. Data are shown as mean ± SD. **C** Imaging (top) and radiance plot (bottom) monitoring tumor growth in donor 879 humanized mice (Donor PBMC-879) treated with control (PBS) or 1B7/CD3 at doses of 1, 0.1, 0.05, and 0.01 mg/kg at indicated time points. Data are shown as mean ± SD. **D** Tumor burden in the bone marrow (BM) from donor 879 humanized mice. BM was harvested 24 h. after the last dosing. Data are shown as mean ± SD; *p < 0.05 compared with control. **E** Percentage of CD3^+^T cells in the blood from donor 879 humanized mice collected at 24 h. post second dosing. Data was analyzed by GraphPad Prism 9 and shown as mean ± SD; **p < 0.01 compared with control. **F** Dynamic shifting of the T cell phenotype as donor 879 humanized mice receive repeated dosing of 1B7/CD3 treatment or control. Blood was collected at week two and week three post treatment. T_SCM_, stem memory T cells (CD45RO^–^CD62L^+^); T_CM_, memory T cells (CD45RO^+^CD62L) ^+^; T_EM_, effector memory T cells (CD45RO^+^CD62L^–^); T_EMRA_, effector memory cells re-expressing CD45RA T cells (CD45RA^+^CD62L^–^).

The in vivo anti-leukemia activity of 1B7/CD3 was first tested in REH-TSLPR-Luc ALL xenograft model. Tumor growth was significantly inhibited in humanized mice treated with 1B7/CD3 when compared to those treated with vehicle (Fig. 3A–B). To confirm the anti-tumor efficacy of 1B7/CD3 in a different donor and assess for dose-dependency, we administered escalating doses of 1B7/CD3 to mice humanized with donor 879. Our findings show that 1B7/CD3 treatment inhibited tumor growth in a dose-dependent manner, with the 1 mg/kg dose inducing the strongest tumor growth inhibition, 0.1 mg/kg inducing moderate growth inhibition, and <0.1 mg/kg inducing no growth inhibition (Fig. 3C). Importantly, nearly complete leukemia clearance in the BM was observed after 1 mg/kg of 1B7/CD3 treatment, resulting in undetectable ALL in 3/5 mice and exceptionally low levels of residual ALL (0.01 and 0.05% of tumor in live cells) in the other two animals (Fig. 3D).

To monitor changes in T cell dynamics, activation, and phenotype, peripheral blood was collected from mice at indicated time points. Consistent with the observed dose-dependent response on tumor growth, the percentage of CD3 significantly increased in mice treated with 1 mg/kg1B7/CD3, but not in other groups, when compared to those treated with vehicle (Fig. 3E). Additionally, 1 mg/kg 1B7/CD3 induced a T cell phenotype shift from effector memory T-cells (T_EM_) to effector memory re-expressing CD45RA T cells (T_EMRA_) as well as an increase in the stem memory T-cells (T_SCM_) population at week 2 and week 3 of treatment when compared to levels among vehicle-treated mice (Fig. 3F).

**Fig. 4:**
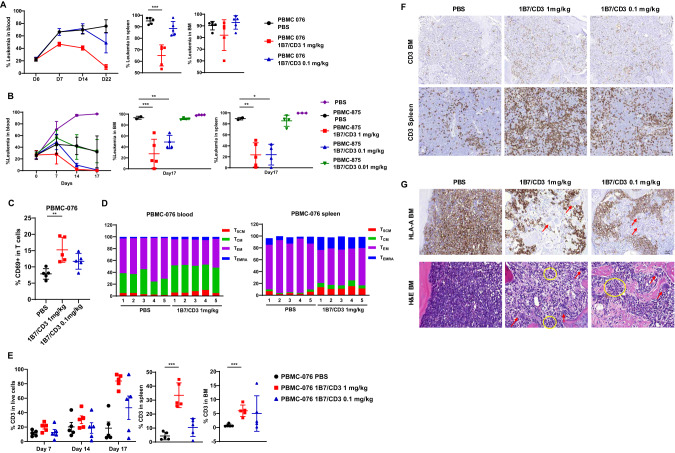
Anti-tumor activity of 1B7/CD3 treatment in the Bos-1 ALL patient-derived xenograft (PDX) model across donors. Three million Bos-1(CD19^+^TSLPR^+^) ALL PDX cells were I.V. injected into NSG mice, and Bos-1 engraftment were assessed by analyzing the percentage of ALL cells (hCD45^+^) in peripheral blood with flow cytometry. Humanization and treatment are same as in Fig. 3A. Leukemia burden was assessed as percentage of hCD45^+^/ TSLPR^+^ cells in single live cell population after humanization. A TSLPR antibody (1B4, BioLegend), non-competing with 1B7/CD3, was used for staining. **A**, **B** Tumor burden, in the blood, spleen, and bone marrow (BM) in (**A**) donor 076 (PBMC 076) and (**B**) 875 (PBMC 875) humanized mice treated with 0.1 or 1 mg/kg 1B7/CD3 or control (PBS). Spleen and BM were harvested 3 days post last dosing. Data are shown as mean ± SD; ***p < 0.001, **p < 0.01, *p < 0.05 compared with control. **C** Percentage of CD69 in CD3 T cells in the blood of donor 076 humanized mice (PBMC 076) treated with 0.1 or 1 mg/kg 1B7/CD3 or control (PBS) at week 2 post dosing. Data are shown as mean ± SD; **p < 0.01 compared with control. **D** Dynamic shifting of the T cell phenotype in the blood and spleen in donor 076 humanized mice (PBMC-076) treated with 1 mg/kg 1B7/CD3 or control (PBS). T_SCM_, stem memory T cells; T_CM_, memory T cells; T_EM_, effector memory T cells; T_EMRA_, effector memory cells re-expressing CD45RA T cells. **E** Percentage of CD3^+^ T cells in the blood, spleen, and BM in donor 076 humanized mice (PBMC-076) treated with 0.1 or 1 mg/kg 1B7/CD3 or control (PBS). Blood samples were collected at indicated times; spleen and BM samples were collected at 3 days post last dosing. Data are shown as mean ± SD; ***p < 0.001 compared with control. **F** Immunohistochemistry (IHC) analysis of CD3 T cells in the spleen and BM in donor 076 humanized mice treated with 0.1 or 1 mg/kg 1B7/CD3 or control (PBS) at 3 days post last dose. **G** HLA-A IHC analysis (upper panel) and hematoxylin & eosin staining in BM (lower panel) in donor 076 humanized mice at 3 days post last dose. Yellow circles indicate mouse polymorphonuclear cells; red arrows indicate mouse megakaryocytes.

Since PDX models have been shown to better recapitulate patient responses, we assessed the anti-ALL activity of 1B7/CD3 treatment in Bos-1 B-ALL xenograft models by FACS analysis. To address the variation of efficacy caused by effector cells from different donors, PBMCs from two donors, 076 and 875, were used for the assessment of anti-tumor efficacy. Overall, 1B7/CD3 treatment induced tumor regression in a dose-dependent manner in the blood, spleen, and BM of both humanized models (Fig. 4A–B). However, and consistent with findings in CDX models, anti-tumor efficacy varied between donors (Fig. 4A–B), with tumor regression observed after 1 and 0.1 mg/kg 1B7/CD3 treatment in the 875 humanized model, but only after 1 mg/kg 1B7/CD3 treatment in the 076 humanized model (Fig. 4A–B).

Treatment-induced effects on T cell dynamics and phenotype were then assessed. When compared to vehicle-treated group, administration of 1 mg/kg, but not 0.1 mg/kg, 1B7/CD3 increased CD69 expression in T cells (Fig. 4C) as well as induced cell phenotype shifting from T_EM_ to T_EMRA_ and increased T_SCM_ populations in both blood and spleen from 076 humanized mice (Fig. 4D). Consistently, a dose and time-dependent increase in T cell concentration was observed in the blood, spleen, and BM of 076 humanized mice treated with escalating doses of 1B7/CD3 when compared to those treated with vehicle, with the strongest increases induced by 1 mg/kg 1B7/CD3 treatment (Fig. 4E). By contrast, and in agreement with previous data (Fig. 4A–B), both 1 and 0.1 mg/kg 1B7/CD3 doses induced strong but similar increases in T cells over time in 875 humanized model when compared to that induced by vehicle (Supplementary Fig. 3A–C). Follow-up IHC analyses confirmed 1B7/CD3 treatment-induced increases in T cells in the BM and spleen of both 076 (Fig. 4F) and 875 humanized mice (Supplementary Fig. 3D). Most importantly, a marked decrease in leukemia cells, and correspondingly, a constitution of normal differentiated mouse myeloid lineage, was observed by H&E staining in the BM of 076 humanized models treated with either 1 or 0.1 mg/kg 1B7/CD3 when compared to that in vehicle-treated groups (Fig. 4G).

### Durable PK in mice

**Fig. 5:**
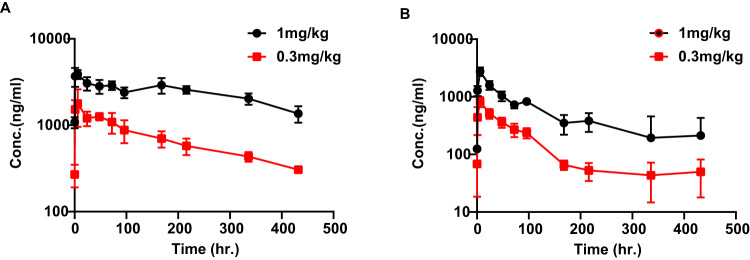
Pharmacokinetics (PK) of the 1B7/CD3. **A**, **B** Pharmacokinetics of a single dose of 1 mg/kg or 0.3 mg/kg 1B7/CD3 in (**A**) NSG and (**B**) humanized NSG mice. Humanized NSG mice were humanized with 10 M human PBMC 10 days before an intraperitoneal (ip) injection of 1B7/CD3, and blood was collected at indicated time points post-injection. through serial sampling and processed to plasma for bioanalytical and PK analyses. Concentrations of 1B7/CD3 in plasma samples were measured using an antigen coated ECL based ELISA, and PK analyses were performed according to standard non‐compartmental analysis using WinNonlin.

Pharmacokinetic analyses of NSG mice treated with 0.3 and 1.0 mg/kg 1B7/CD3 revealed a durable PK profile in serum and an approximate half-life of 9-10 days (Fig. 5A; Supplementary Table 1). This approximate half-life is similar to that of normal IgG antibody and markedly longer than the reported half-life of blinatumomab [24] (Fig. 5A; Supplementary Table 1). The potential for target-mediated drug disposition (TMDD) during the binding of the CD3 arm of the 1B7/CD3 to human T cells was then assessed with a second PK study using serum harvested from PBMC humanized NSG mice. Compared to findings in NSG mice, 1B7/CD3 demonstrated a relatively shorter half-life of 4–5 days and lower Cmax in humanized NSG mice (Fig. 5B; Supplementary Table 2), suggesting that 1B7/CD3 would possibly exhibit TMDD in patients.

### Endurable safety in cynomolgus monkeys

To determine whether cynomolgus monkeys were the most appropriate models for predicting the toxicity of 1B7/CD3 treatment in patients, we first demonstrated that the binding properties of cynomolgus monkey PBMCs to anti-TSLPR and the BsAb of 1B7/CD3 were similar to those of human PBMCs. Anti-TSLPR antibodies showed negligible or no binding to T cells (Supplementary Fig. 4A), hCD14, hCD16, hCD20, and hCD56 positive populations (data not shown). While consistent with literature [25], there is a detectable expression of TSLPR in DCs derived from human monocytes but not B cells from cynomolgus monkey BM (Supplementary Fig. 4B, C). In contrast, 1B7/CD3 bound to T cells gated as CD3^+^ PBMC, thus indicating that the CD3 arm of the BsAb binds properly to both human and cynomolgus monkey T cells (Supplementary Fig. 4A).

**Fig. 6:**
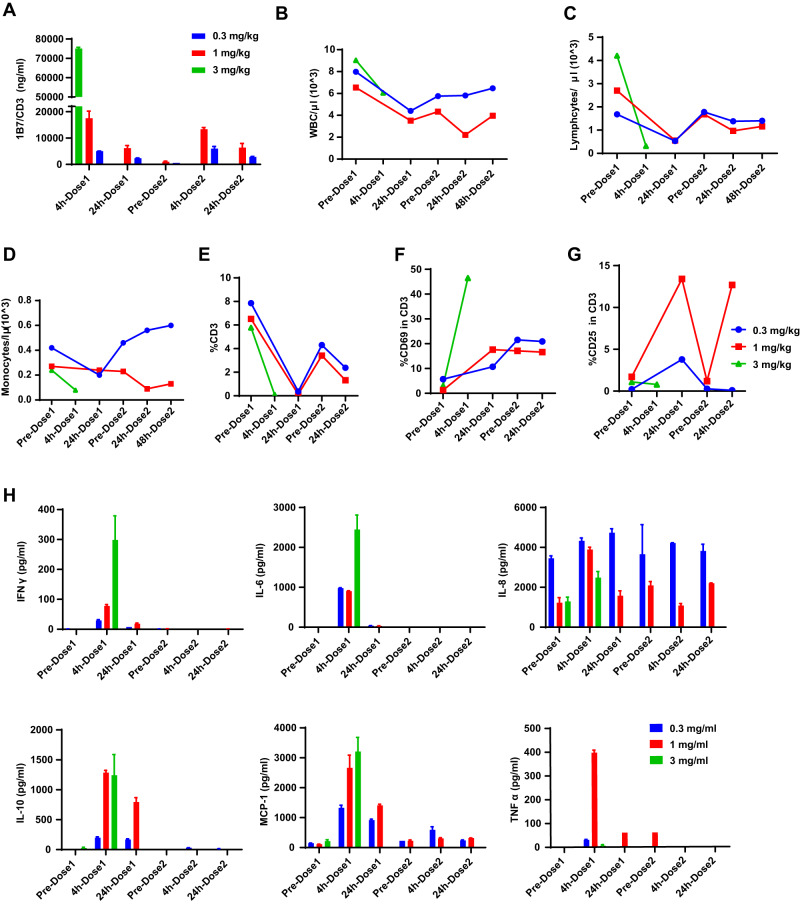
Toxicity evaluation of the safety of 1B7/CD3 treatment in cynomolgus monkey. Three adult, female cynomolgus monkeys were randomized into treatment groups and received 0.3, 1, or 3 mg/kg 1B7/CD3 via iv bolus on Day 1 and Day 8. Monkeys were observed at least twice daily for moribundity and mortality and were monitored closely for the first four hours following each dose administration. **A** 1B7/CD3 plasma exposure at indicated time points in cynomolgus monkeys treated with 0.3, 1, or 3 mg/kg 1B7/CD3. Data are shown as mean ± SD. **B**–**D** Concentrations of (**B**) white blood cells (WBC), (**C**) lymphocyte, and (**D**) monocyte at indicated time points in cynomolgus monkeys treated with 0.3, 1, or 3 mg/kg 1B7/CD3. Cell counts were performed by a hematological analyzer. **E** Dynamic change in CD3 positive T cells in the blood collected at indicated time points in cynomolgus monkeys treated with 0.3, 1, or 3 mg/kg 1B7/CD3. **F**, **G** Dynamic changes of (**F**) CD69 and (**G**) CD25 expression in T cells in the blood collected at indicated time points in cynomolgus monkeys treated with 0.3, 1, or 3 mg/kg 1B7/CD3. **H** Induction of cytokine levels by 0.3, 1, or 3 mg/kg 1B7/CD3 treatment in cynomolgus monkeys at indicated time points.

An exploratory toxicity study was then conducted to evaluate the safety of 1B7/CD3 in vivo. Initially, doses of 3, 1, or 0.3 mg/kg were evaluated in cynomolgus monkeys, but the highest dose caused progressive lethargy and resulted in euthanasia 4 h after the first dose. Doses of 1 and 0.3 mg/kg resulted in appropriate antibody exposure, with the second dose of 1 or 0.3 mg/kg reaching a Cmax similar to or slightly lower than its respective initial dose. Additionally, antibody concentration declined in half 24 h post-dosing. Notably, the plasma concentration of 1B7/CD3 after receiving an initial dose of 0.3 mg/kg was found to be 536 ng/ml on the day before second dosing (Fig. 6A), which is still well above the picomolar range EC_50_ observed in vitro (Fig. 6A). Importantly, the 1 mg/kg dose was tolerated with mild and transient treatment-related adverse events (AE), and the 0.3 mg/kg dose was well-tolerated with minor treatment-related AEs. Specifically, for both 1 and 0.3 mg/kg dosage groups, white blood cell, monocyte, and lymphocyte counts briefly declined after the first dosing and gradually rebounded once the dosing was stopped (Fig. 6B–D). Follow-up histopathological analysis found no lesions that could be specifically attributed to 1B7/CD3 treatment in cynomolgus monkeys. Together, these data suggest that AEs associated with 1B7/CD3 treatment are dose-dependent.

Consistent with the hematology data, we observed a steep decrease in T cells at 24 h post first dose and, to a lesser extent, at 24 h post second dose, followed by a modest rebound of T cell quantity at 7 days after dosing in cynomolgus monkeys treated with 1B7/CD3 (Fig. 6E). Correspondingly, the CD69 percentage in T cells increased significantly after the first dose and remained at a steady state without further rise after the second dose of 1 or 0.3 mg/kg 1B7/CD3 (Fig. 6F). Of note, a rapid decline of T cell percentage and a rapid increase in the CD69 percentage in T cells were observed in the cynomolgus monkey treated with 3 mg/kg (Fig. 6E–F). Additionally, a sharp and transient increase in the expression of the T cell activation marker, CD25, was observed 24 h after the first and second doses of 1 mg/kg 1B7/CD3, with levels returning to baseline levels at 7 days post-dosing (Fig. 6G). While moderate increase 24 h after the first dose of 1B7/CD3 at 0.3 mg/kg, but not after the second dose (Fig. 6G). Lastly, and in line with clinical observations, serum samples from cynomolgus monkeys that received 3, 1, or 0.3 mg/kg 1B7/CD3 exhibited transient increases of IFNγ, IL-6, IL-8, IL-10, monocyte chemoattractant protein 1 (MCP-1), and TNFα cytokines at 4 h post initial dose and a return to basal levels at either 24 h or 7 days post dosing. No notable induction of cytokine levels was observed after second dosing, except for IL-8 (Fig. 6H).

## Discussion

To date, more than 100 different BsAb formats exist, with new constructs constantly emerging due to efforts to extend the half-life and optimize the balance between anti-tumor activity and drug safety [26–28].

Here, we present 1B7/CD3, a novel anti-TSLPR BsAb comprised of TSLPR 1B7 Fab and Xencor’s anti-CD3-scFv. In vitro analysis demonstrated proper binding of the 1B7/CD3 to both human TSLPR and CD3 with high purity and feasible antibody production. Interestingly, while TSLPR is primarily expressed on monocytes and dendritic cells, as well as occasionally on lymphocytes [29, 30], 1B7/CD3 induced only minimal activation of normal PBMC but strong antigen-specific T cell activation and potent tumor cell killing. Follow-up in vivo assessments found that 1B7/CD3 treatment markedly inhibited tumor growth in distinct CDX and PDX models across donors at doses that were tolerable to cynomolgus monkeys. More PBMC donors will be included in future studies to further address the donor to donor variation.

Remarkable in vivo anti-ALL activity of 1B7/CD3 was associated with T cell expansion in the spleens and BM. We also showed 1B7/CD3 induced phenotype shift from T_EM_ not only to T_EMRA_ but also to T_SCM_, which have the ability to self-renew and proliferate. The presence of mouse polymorphonuclear cells and megakaryocytes was observed in the BM from 1B7/CD3-treated mice by H&E analysis, which indicates that IB7/CD3 can selectively kill leukemia cells but spare normal hematopoietic cells. Together, our data demonstrated that the exceptional tumor growth inhibition of 1B7/CD3 therapy was likely mediated by T cell activation and expansion.

The 1B7/CD3 BsAb has been designed to address the short half-life of blinatumomab, which is due to its lack of an Fc domain. Specifically, our 1B7/CD3 BsAb includes Fc to facilitate purification, enhance stability, and prolong the half-life by FcRn binding [31]. Consequently, 1B7/CD3 maintains full-length antibody properties and a half-life of about 9–10 days in NSG mice, similar to that of a typical monoclonal antibody in humans [32]. Follow-up efforts focused on assessing the possibility of TMDD in patients treated with 1B7/CD3 by using PBMC humanized NSG mice. A target sink effect has been reported in daratumumab, a CD38 monoclonal antibody, due to the abundant expression of CD38 in myeloma [33, 34]. Given that BsAbs bind two targets simultaneously, the TMDD effect may be exacerbated once both targets are present. Hence, the PK profile of 1B7/CD3 in NSG mice may not reflect the PK profile in patients since 1B7 does not cross binding to the mouse and NSG mice lack T cells. As expected, assessment of the PK profile of 1B7/CD3 in humanized NSG mice revealed a reduction of Cmax and half-life. Further, the concentration of 1B7/CD3 in the distribution phase was profoundly reduced in humanized mice when compared to that in wild-type mice, which was possibly caused by the CD3-mediated antibody sink. Consistently, this profound decline in 1B7/CD3 exposure was also observed in the cynomolgus monkey at 24 h post-doses. However, it is important to note that the half-life of 1B7/CD3 is still significantly longer than that of blinatumomab, and the serum exposure of 1B7/CD3 at 18 days post dosing is higher than the EC_50_ in in vitro killing assay.

Dosing-limiting toxicities have been observed in the majority of CD3-redirecting BsAbs, including blinatumomab, in both patients and preclinical models, with the most common AEs associated with cytokine release syndrome (CRS) and neurotoxicity [35, 36]. Cynomolgus monkey is an appropriate model for assessing 1B7/CD3 toxicity, as 1B7/CD3 binds to human and cynomolgus TSLPR and CD3 with comparable affinities. In cynomolgus monkey, we found that a high dose (3 mg/kg) of 1B7/CD3 induced AEs such as progressive vomiting and lethargy. However, medium (1 mg/kg) and low (0.3 mg/kg) doses of 1B7/CD3 were well-tolerated with only mild and transitory BsAb-related toxicity. Importantly, there is appropriate antibody exposure over the period of toxicity assessment. Administration of 1B7/CD3 induced a dose-dependent depletion of lymphocytes and monocytes at 4 h after the first dose, likely caused by activation-induced T cell death [37], with a full or partial recovery at 24 h or 7 days after the first dose. We did not observe a reduction in leukocytes after the second dose in cynomolgus monkeys, which is consistent with reports from other BsAbs. Interestingly, we observed a marked elevation of cytokines after the first dosing, but not after the second dose, with the exception for IL-8. A transient but strong elevation of pleiotropic cytokines within minutes to hours after an infusion of CD3-redirecting BsAbs has been previously observed, as well as shown to be likely mediated by helper T cells and macrophages [38, 39]. Further, a strong elevation of cytokines, primarily TNF-a, interferon (IFN)-g, IL-1b, IL-2, IL-6, IL-8, MCP-1, and IL-10, have been implicated in the pathogenesis of CRS [40, 41]. Together, our assessment of 1B7/CD3 toxicity in cynomolgus monkeys strongly indicated that the maximum tolerated dose of our CD3-redirecting BsAb is 1 mg/kg. However, more animals will be needed in future GLP toxicity study.

Collectively, our preclinical studies demonstrate potent anti-B-ALL activity of 1B7/CD3 in vitro and in vivo at doses that are tolerable in cynomolgus monkeys. These findings provide the proof of concept for evaluating BsAb-targeting TSLPR as a potential therapy for improving the outcome for patients with CRLF2/TSLPR-overexpressing Ph-like B-ALL.

### Supplementary information

Supplemental material
