# Correction: Preclinical development of 1B7/CD3, a novel anti-TSLPR bispecific antibody that targets *CRLF2*-rearranged Ph-like B-ALL

**DOI:** 10.1038/s41375-023-02090-w

**Published:** 2023-11-30

**Authors:** Ze Tian, Chunhua Shi, Guojun Yang, Jason K. Allen, Qing Shi, Amin AL-Shami, Jill Wardell Olson, Melinda G. Smith, Qing Chang, Jasbir Kaur, Junping You, Timothy E. Lofton, Michelle A. Gonzalez, Qi Zhang, DongXing Zha, Sarah K. Tasian, Nitin Jain, Marina Y. Konopleva, Timothy Heffernan, Jeffrey J. Molldrem

**Affiliations:** 1 ORBIT Platform, The University of Texas MD Anderson Cancer Center, Houston, TX USA; 2 Department of Leukemia, Division of Cancer Medicine, The University of Texas MD Anderson Cancer Center, Houston, TX USA; 3 Division of Oncology and Center for Childhood Cancer Research, Children’s Hospital of Philadelphia; Department of Pediatrics and Abramson Cancer Center, University of Pennsylvania School of Medicine, Philadelphia, PA USA; 4 Translational Research to Advance Therapeutics and Innovation in Oncology (TRACTION), The University of Texas MD Anderson Cancer Center, Houston, TX USA; 5 Department of Hematopoietic Biology & Malignancy, Division of Cancer Medicine, The University of Texas MD Anderson Cancer Center, Houston, TX USA

**Keywords:** Cancer immunotherapy, Immunotherapy

Correction to: *Leukemia* 10.1038/s41375-023-02010-y, published online 26 August 2023

In this article the author’s name Amin AL-Shami was incorrectly written as Amin AI-Shami.

The original article has been corrected.

