# Supplementary material for: Preclinical development of 1B7/CD3, a novel anti-TSLPR bispecific antibody that targets CRLF2-rearranged Ph-like B-ALL
Source: Leukemia. 2023 Aug 26;37(10):2006–16. doi: 10.1038/s41375-023-02010-y (PMC10539166; doi:10.1038/s41375-023-02010-y)
Supplement: Supplementary file 1 — Supplemental material [file 41375_2023_2010_MOESM1_ESM.pdf]

Supplemental methods

Immunization and Antibody Titer measurement

H2L2 female mice were purchased from Harbour BioMED. Blood was collected from the 10-week-old mice for baseline measurements. A subcutaneous immunization consisting of 100 µl of a 1:1 (v: v) mix of RIBI adjuvant (S6322-1VL, Sigma) and 50 µg recombinant TSLPR-Fc fusion proteins in PBS was then administered to each mouse at the groin region. The mice were subsequently administered 6 rounds of booster doses on a biweekly schedule using the same regimen. Titer was measured at 3 days after the 5th booster dose. The final booster, consisting of 200 µl, was administered intraperitoneally 4 days after the 5th booster dose. Mice were euthanized 4 days after the final booster dose, and the spleen was harvested for antigen-specific memory B-cell sorting.

96-well EIA high bind plates (Costar #3361) were coated with 2 µg/mL of TSLPR, Fc tag (R&D Systems #981-TR-050) at 4°C overnight. Plates were blocked with 1% non-fat dry milk plus 1% BSA in PBS containing 0.05% Tween-20 (PBS-T) for 30 minutes at room temperature (RT). Plates were then incubated with serial dilutions of mouse serum in blocking buffer for 1 hr at RT. After washing, bound antibody was detected by incubation with HRP-conjugated horse anti-Rat IgG (Cell Signaling #7077) for 1 hr at RT. Plates were washed again, and then developed using TMB substrate (Thermo Fisher Scientific). The reaction was stopped

using H_2SO_4 and absorbance was measured at 450 nm using a CLARIOstar® Plus plate reader (BMG Labtech).

Single B-cell Cloning (SBCC) of the anti-TSLPR variable region

H2L2 Harbour mice were used to develop the fully human anti-TSLPR antibody. After 10 weeks of immunization with human TSLPR-Fc fusion proteins, single B-cells from spleen were sorted with a panel of biomarkers. After RT-PCR was used to generate cDNA, Harbour H2L2 heavy (18) and light (11) degenerative forward primers, together with rat constant region reverse primers, were used to amplify the variable regions. These variable regions were subsequently cloned into a pcDNA3.1+ expression vector for high throughput transient expression of ExpiCHO cells using a 96-well format.

High throughput monoclonal antibody (mAb) production, purification, and analysis

High-throughput production of mAbs was performed by micro-scale transfection (1 mL) of pcDNA3.1+ antibody expression vectors in Chinese hamster ovarian (CHO) cells using the Gibco™ ExpiCHO™ Expression System and a protocol for deep 96-well plates (ThermoFisher Scientific). Briefly, synthesized antibody-encoding DNA (0.8 µg per transfection) was diluted in OptiPro serum free medium (OptiPro SFM), incubated with ExpiFectamine CHO Reagent, and added to 750 µL of ExpiCHO cell cultures into sterile 96 deep well plates using a ViaFlo 96 liquid handler (Integra

Biosciences). Plates were placed in an Infors HT Multitron Pro incubator shaking at 1,000 rpm with 3 mm orbital diameter at 37°C in 8% CO2 and 80% humidity. The day after transfection, ExpiFectamine CHO Enhancer and ExpiCHO Feed reagents were added to the cells, followed by a 6-day incubation at 32°C in 5% CO2 and 80% humidity.

Cells were harvested by centrifugation at 1500 x g for 10 min and supernatants were transferred to new deep 96-well plates for high throughput micro-scale purification. Briefly, clarified culture supernatants were incubated with 15 µL MabSelect resin (Cytiva, formerly GE Healthcare Life Sciences) on a 3 mm orbital shaker at 1000 rpm at RT to capture mAb. The mixture was then transferred to pre-equilibrated fritted deep well filter plates, washed with PBS, and eluted with 100 µL 50mM phosphoric acid (pH 3.0) into 96-well plates containing 15 µL neutralization buffer (20X PBS pH 11.0).
Recovered fractions were analyzed for yield, size, and purity using the LabChip® GXII HT Touch™ (Perkin Elmer, CLS138160) microfluidic CE-SDS platform and the ProteinEXact Reagent Kit (Perkin Elmer, CLS150466) according to the manufacturer's protocol. Briefly, 2.5 µl of purified protein was mixed with 18 µl of sample buffer and 8.75 mM Iodoacetamide (Thermo Fisher Scientific, A39271) for non-reducing conditions. Samples were then heated to 70°C for 10 min, cooled to RT, and mixed with 35 µl of water. The analysis was performed using GXII Reviewer software and calculations were based on the internal reference ladder.

Large scale mAb production and purification

For large scale mAb expression, we performed transient transfections of CHO cell cultures using the Gibco™ ExpiCHO™ Expression System in Erlenmeyer vented cap flasks (Corning) or single-use ReadyToProcess WAVE Cellbags (Cytiva) containing the desired volume of ExpiCHO cells following the manufacturer's protocol. A 1:1 ratio of heavy and light chain plasmids were transfected for IgG format antibodies while a 1:2:1 ratio of heavy:light:CD3 scFv plasmids were transfected for bispecific antibodies. Antibodies were purified from filtered culture supernatants by fast protein liquid chromatography (FPLC) on an ÄKTA Pure instrument using a HiTrap MabSelect PrismA column (Cytiva). IgG format antibodies were eluted by 20mM sodium citrate (pH 3.0) and bispecific format antibodies were eluted with 0.5M arginine (pH 4.0). Purified mAbs were concentrated and buffer exchanged into PBS using Amicon® Ultra 50KDa Centrifugal Filter Units (Millipore Sigma), filtered using sterile 0.2 µm pore size filter devices (Millipore), and stored in aliquots at 4°C until future use.

MAb quantification

To quantify purified mAbs, absorption at 280 nm (A280) was measured using a NanoDrop (ThermoFisher Scientific). mAb

concentration was calculated using the IgG sample type setting, which uses a typical molar extinction coefficient for IgG, on the NanoDrop.

Biolayer Interferometry (BLI) for Anti-TSLPR monoclonal antibody screening

BLI assays were performed on the Octet Red384 instrument at 30ºC with shaking at 1,000 RPM. The mAb binding screen was performed after 96-well high-throughput expression and protein A purification. The different clones of mAbs were diluted in PBS to a concentration of 100nM (15µg/ml) and captured using anti-human IgG Fc capture (AHC) biosensors for 600 seconds. After loading, the baseline signal was then recorded for 60 seconds in 10xkinetics buffer from the vendor. The sensors were then immersed in 10xkinetics buffer containing 100, 20, or 4nM TSLPR-His recombinant protein for a 300 second association step. The dissociation was then measured for 300 seconds by immersing sensors in 10xkinetics buffer. As a control for non-specific binding, the background signal of empty sensors without loading antibodies was subtracted at each time point.

Kinetic analyses

For kinetic analyses, either 1B7 or 1B7/CD3e bispecific antibodies was captured using AR2G sensors; ligands were diluted to 100 nM in 10xkinestics and loaded for 600 seconds. After the loading

and quenching of the active AR2G surface by 1M ethanolamine, the baseline signal was then recorded for 1 min in 10xkinetics.

Binding of anti-TSLPR and 1B7/CD3 antibodies to human and cynomolgus monkey peripheral blood mononuclear cells (PBMCs)

Binding of anti-TSLPR IgG and 1B7/CD3 bispecific antibodies were analyzed using PBMCs from at least three different healthy individuals and cynomolgus monkeys. Human PBMCs were isolated from buffy coat and cynomolgus PBMCs were isolated from whole blood (KCCMR). Isolated PBMCs were stained with either anti-TSLPR or 1B7/CD3 together with the following leukocyte phenotype markers: CD3 (BD pharmingen), CD4, CD8, CD14, CD16, CD20 and CD56 (Biolegend). Data were acquired by flow cytometry and analyzed using FlowJo as described previously.

Generation and maturation of human monocyte-derived dendritic cells

PBMCs were isolated by Ficoll-Hypaque gradient centrifugation from healthy donors. Monocytes were purified with a CD14 isolation kit following the manufacturer’s protocol (Miltenyi Biotec, Bergisch Gladbach, Germany). Purified monocytes were cultured

in RPMI with 10% FBS, 50uM beta-mercaptoethanol, hGM-CSF (100ng/mL, R&D), and hIL-4 (100ng/mL, R&D) for 5-7 days and subsequently matured with TNF-α for 24 hours. TSLPR expression of both immature and matured dendritic cells was tested by flow cytometry with anti-TSLPR-AF647.

Supplemental Figure legends

Supplemental Figure 1. Development of anti-TSLPR monoclonal antibodies (mAbs).

(A) Schematic of immunization in human transgenic mice. H2L2 Harbour human transgenic mice were immunized for 10 weeks with hTSLPR-Fc antigen, and the spleen was harvested. **(B)** Single cell sorting of antigen-specific memory B cells. mCD19 and hTSLPR-positive cells were sorted for single B-cell cloning (SBCC). **(C)** Single cell RT-PCR. Example of single cell reverse transcription (RT) and PCR of TSLPR variable heavy (VH) and variable light (VL) gene regions. **(D-E)** mAbs isolated from SBCC were tested for binding to the TSLPR antigen. (D) Affinity statistics and (E) the correlation between Kon and Koff were plotted using Bio-Layer Interferometry. (**F**) Octet analysis shows blockage of TSLP binding to TSLPR by anti-TSLPR mAbs. (**G**) Germline distribution of all the discovered anti-TSLPR mAbs, as calculated by IgBlast. (**H**) Representative epitopes binnings were shown to

indicate the group of bins by both competing antibodies and TSLP ligand blockage.

Supplemental Figure 2. Cytotoxicity of 1B7/CD3 in REH cells.

(A) REH cells with high, medium, or low TSLPR expression and a luciferase marker were incubated with different concentrations of 1B7/CD3 bispecific or control antibodies. PBMC-derived human $CD8^{+}$ cells were then added at a 5:1 ratio (E: T), and flow cytometry was used to determine cell viability after 48 hr. (B and C) AF750 tagged human T cells (B) or BM cells (C) were incubated with different concentrations of 1B7/CD3 BsAb or control antibody before adding untagged effector T cells at a 5:1 ratio (E: T). Flow cytometry was used to determine the cell viability after 48 hr.

Supplemental Figure 3. Pharmacodynamic changes upon 1B7/CD3 treatment in donor 875 PBMC humanized BOS-1 patient-derived xenograft (PDX) models.

(**A-C**) Percentage of $CD3^{+}$ T cells in (A) blood, (B) spleen, and (C) the bone marrow (BM) of donor 875 humanized (PMBC-875) mice treated with 0.01, 0.1, or 1 mg/kg 1B7/CD3 or control (PBS). Samples were collected at either (A) indicated time points or (B-C) at end of experiment. Data analyzed by GraphPad Prism 9 and are shown as mean ± SD; **p< 0.01, *p<0.05 compared with control. (**D**) Immunohistochemistry analysis of CD3 in the BM or

spleen and of HLA-A in the BM in donor 875 humanized mice (PMBC-875) at the end of the efficacy study.

Supplemental Figure 4. Binding pattern of anti-TSLPR (1B7) IgG1 and 1B7/CD3 bispecific antibodies (BsAb) to human and cynomolgus monkey PBMC (T cells).

(A) Flow cytometry analysis showing the binding of 1B7 IgG and 1B7/CD3 BsAbs to PBMCs from at least three different healthy individuals (human) or from cynomolgus monkeys. Isolated PBMCs were stained with either anti-TSLPR or 1B7/CD3 BsAbs with the following leukocyte phenotype markers: CD3, CD4, CD8, CD14, CD16, CD20 and CD56. Representative figures of 1B7 IgG1 and 1B7/CD3 staining are shown in CD3 positive T cell population. Isotype is shown in blue, and 1B7 and 1B7/CD3 staining are shown in red. **(B)** Monocyte-derived DCs were cultured with (right panel) or without (left panel) TNFα for 24 hours before TSLPR expressions were determined by flow cytometry with 1B7-AF647. An isotype control-AF647 antibody was used to set up the positive gate. **(C)** Normal cynomolgus BM cells were collected and stained for CD20 and 1B7 (red) or isotype control (blue). Expression of TSLPR on CD20+ cells were shown in the histogram figure.

Suppl. Figure 1

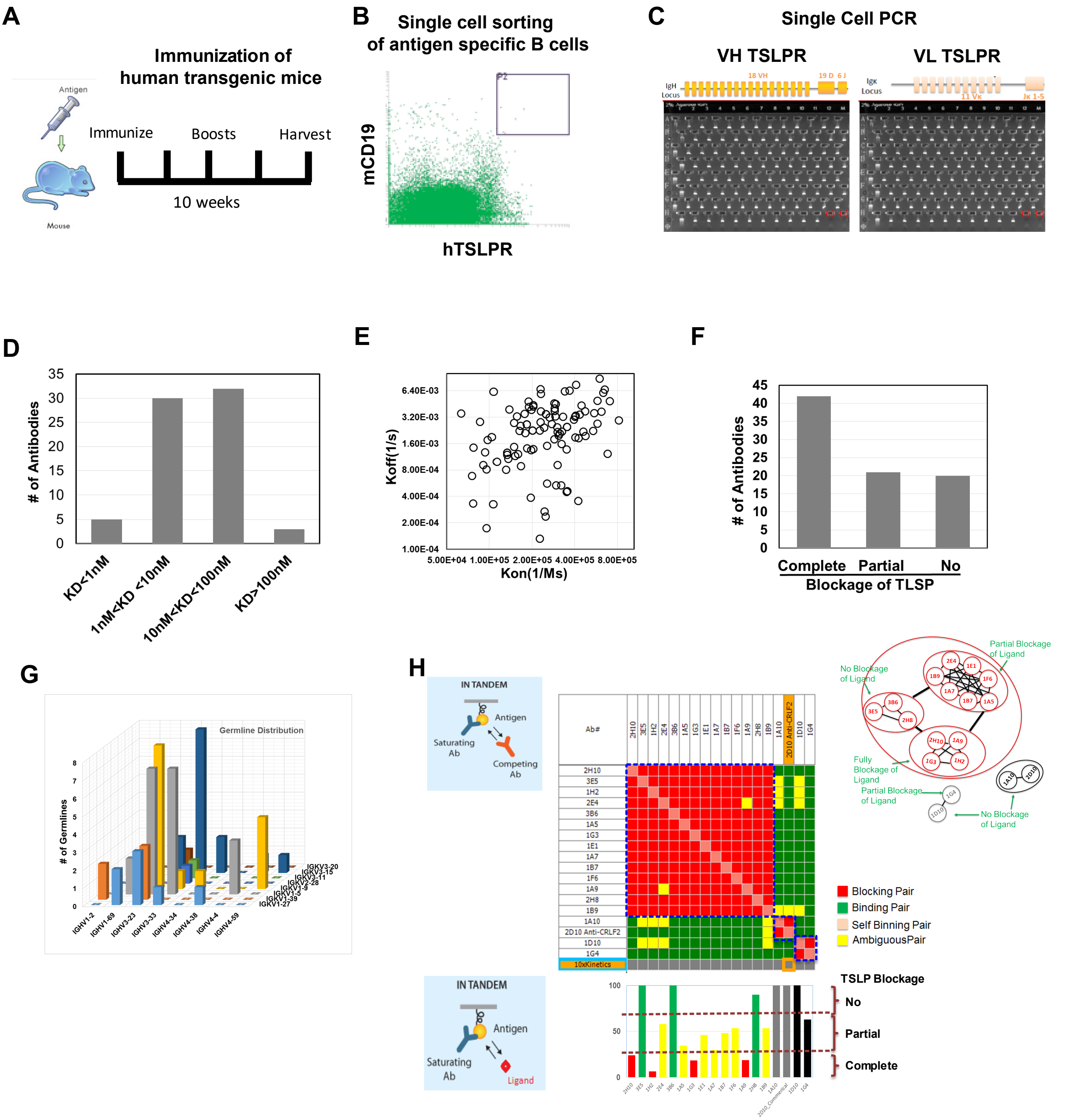

Suppl. Figure 2

A

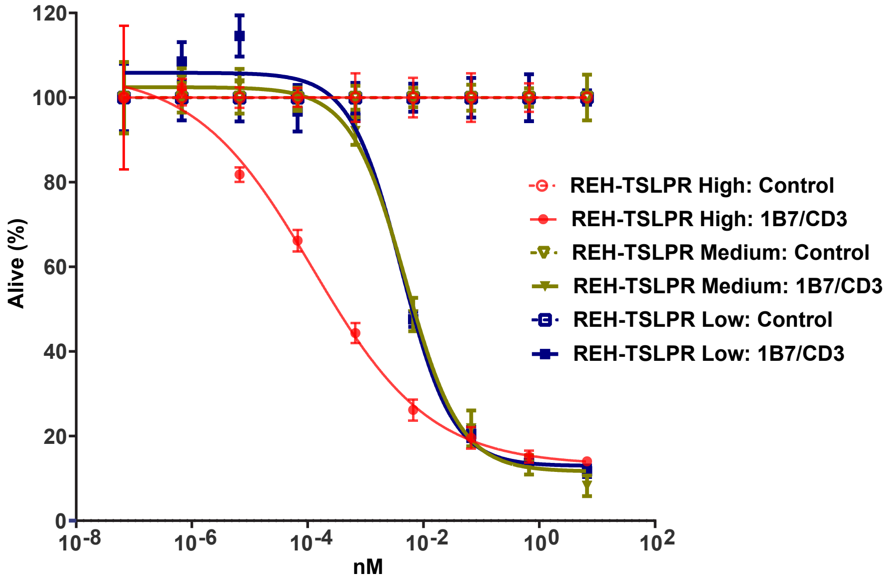

B

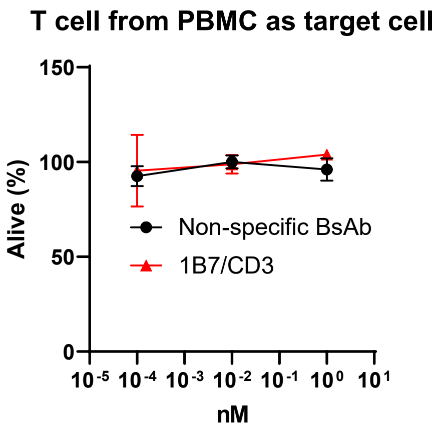

C

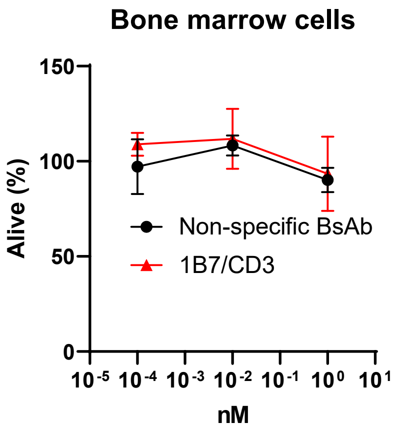

Suppl. Fig 3

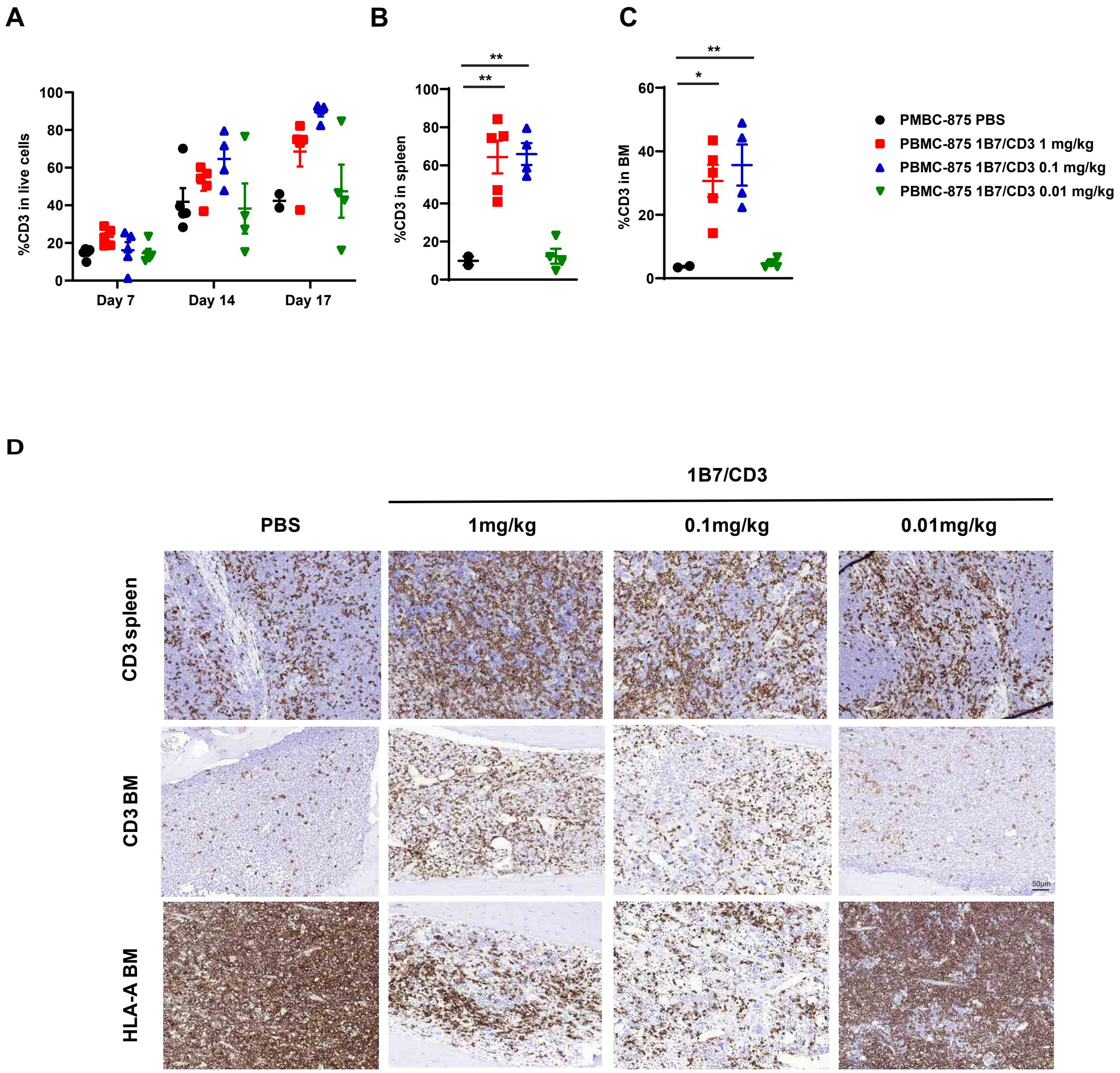
A
B
C
%CD3 in live cells
100
80
60
40
20
0
Day 7
Day 14
Day 17
%CD3 in spleen
**
**
%CD3 in BM
**
*
PMBC-875 PBS
PBMC-875 1B7/CD3 1 mg/kg
PBMC-875 1B7/CD3 0.1 mg/kg
PBMC-875 1B7/CD3 0.01 mg/kg
D
1B7/CD3
PBS
1mg/kg
0.1mg/kg
0.01mg/kg
CD3 spleen
CD3 BM
HLA-A BM

Suppl. Figure 4

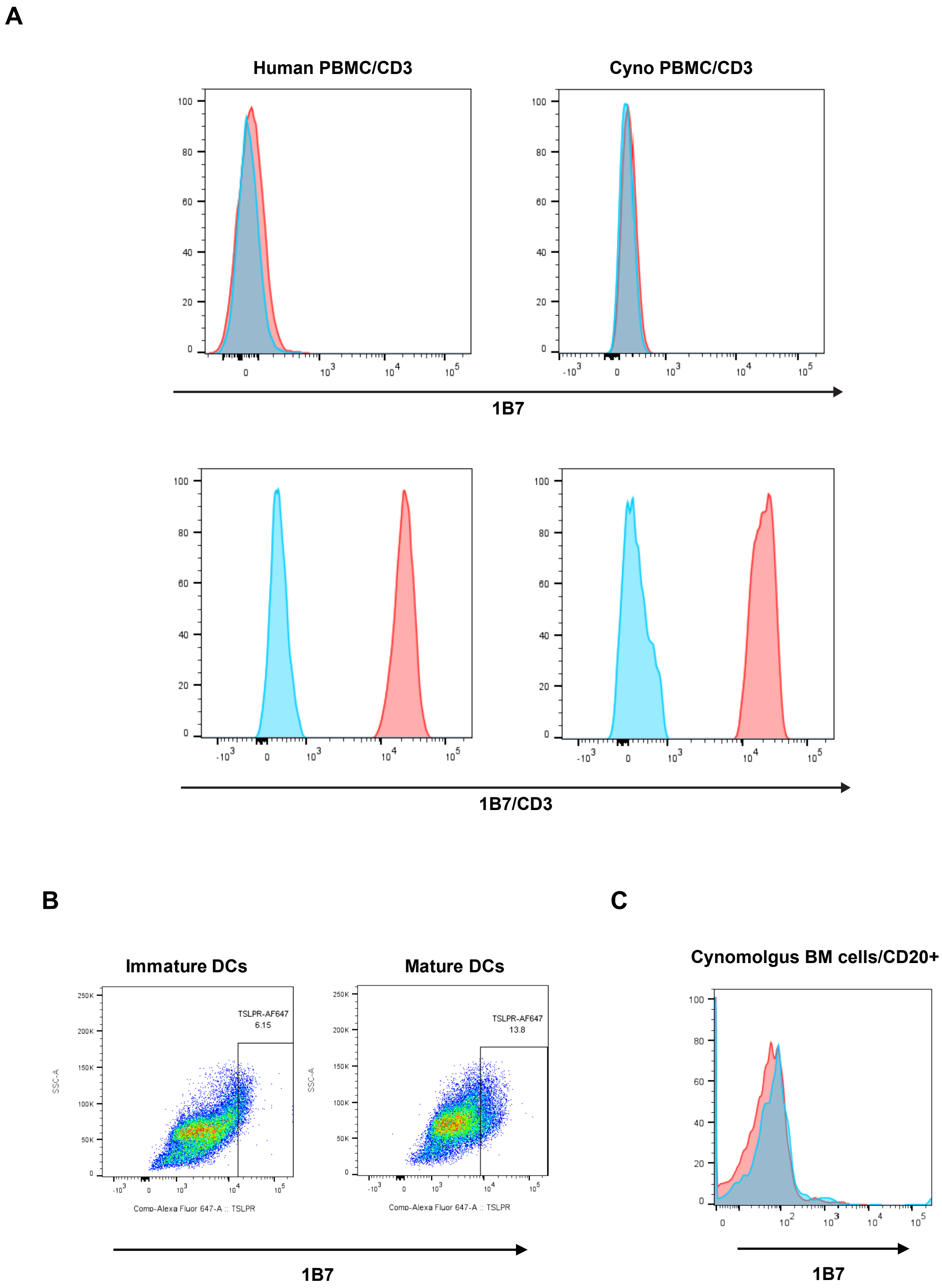

Suppl.table 1-2

Table 1. Pharmacokinetics of 1B7/CD3 BsAb in NSG mice at 1 and 0.3 mg/kg

Dosing (mg/kg)	Lambda_z (1/hr)	T1/2 (hr)	Tmax (hr)	Cmax (ng/ml)	AUCINF_obs (hr*ng/ml)	Vz_obs (ml/kg)	Cl_obs (ml/hr/kg)
1	0.00299±0.000393	234.79±30.506	4.333±2.887	4019.33±674.579	1506434±292201	0.00023±2.86E-05	6.8E-07±0.00000012
0.3	0.003±0.001	227.3±35.784	10.33±12.1	1890±744.85	398447±32875.668	0.00025±0.0000584	7.6E-07±0.00000006

Table 2. Pharmacokinetics of 1B7/CD3 BsAb in humanized NSG mice at 1 and 0.3 mg/kg

Dosing (mg/kg)	Lambda_z (1/hr)	T1/2 (hr)	Tmax (hr)	Cmax (ng/ml)	AUCINF_obs (hr*ng/ml)	Vz_obs (ml/kg)	Cl_obs (ml/hr/kg)
1	0.007±0.003	122.639±70.156	6.000±0	2764.120±2764.120	283064.903±139971.589	613.600±104.418	4.050±1.339
0.3	0.007±0.002	99.510±25.321	6.000±0	827.298±163.509	69870.336±19523.340	626.750±97.793	4.582±1.403

Cmax, maximum (peak) concentration [ng/mL]; tmax, time to reach Cmax [hr]; t1/2, elimination half-life [hr]; AUC, area under the curve [hr*ng/mL]; CL, clearance [mL/hr/kg]; Vz, volume of distribution [mL/kg]; F, female; M, male; N, number of animals; SD, standard deviation.

Supplementary Table 3 -- TISSUE COLLECTION AND PRESERVATION				
TISSUE	WEIGH	COLLECT	MICROSCOPIC EVALUATION	COMMENT
Artery, aorta	-	x	x	-
Bone, femur	-	x	x	-
Bone, sternum	-	x	x	-
Bone marrow, femur	-	x	x	-
Bone marrow, sternum	-	x	x	Five brain levels to be examined
Brain[a]	x	x	x	-
Esophagus	-	x	x	-
Eye, left	-	x	x	Preserve in modified Davidson's fixative.
Eye, right	-	x	x	Preserve in modified Davidson's fixative.
Gallbladder	-	x	x	-
Gland, adrenal, left	x	x	x	-
Gland, adrenal, right	x	x	x	-
Gland, mammary	-	x	x	For males, examine only if present in routine section of skin.
Gland, parathyroid, left	-	x	x	Examine only if present in the routine section of thyroid.
Gland, parathyroid, right	-	x	x	Examine only if present in the routine section of thyroid.
Gland, pituitary	x	x	x	-
Gland, salivary	-	x	x	Both collected; only 1 required for microscopic examination.
Gland, thyroid, left	x	x	x	Weight includes parathyroid
Gland, thyroid, right	x	x	x	Weight includes parathyroid
Gross lesion	-	x	x	-
Gut-associated lymphoid tissue	-	x	x	Examine only if present in routine section of intestines.
Heart	x	x	x	-
Kidney, left	x	x	x	-
Kidney, right	x	x	x	-
Large intestine, cecum	-	x	x	-
Large intestine, colon	-	x	x	-
Large intestine, rectum	-	x	x	-
Liver	x	x	x	Draine gallbladder before weighing.
Lung	-	x	x	-
Lymph node, mandibular	-	x	x	Only 1 required for examination.
Lymph node, mesenteric	-	x	x	Only 1 required for examination.
Muscle, quadriceps femoris	-	x	x	-
Nerve, optic, left	-	x	x	Examine only if present in the routine section of the eye. Preserve in modified Davidson's fixative.
Nerve, optic, right	-	x	x	Examine only if present in the routine section of the eye. Preserve in modified Davidson's fixative.
Nerve, sciatic	-	x	x	Both collected; only 1 required for microscopic examination.
Ovary, bilateral	-	x	x	Paired examination.
Pancreas	-	x	x	-
Site, injection	-	x	x	Location: peripheral vein, as documented in NONHUMAN PRIMATE DOSE FORM (KCCMR Form 224)
Skin	-	x	x	Location: chest
Small intestine, duodenum	-	x	x	-
Small intestine, ileum	-	x	x	-
Small intestine, jejunum	-	x	x	-
Spinal cord	-	x	x	Examine one transverse and one oblique section from each of the following areas: cranial cervical, mid-thoracic, caudal lumbar.
Spleen	x	x	x	-
Stomach	-	x	x	-
Thymus	-	x	x	-
Tongue	-	x	x	-
Trachea	-	x	x	-
Urinary bladder	-	x	x	-
Uterus	-	x	x	-
Vagina	-	x	x	-

x=Procedure to be conducted; - = Not applicable

[a] The brain will be sectioned generally according to the recommendations in the Society of Toxicologic Pathology (STP) Position paper regarding sampling the nervous system (Bolon et al., 2013), but with the modification that level 1 (olfactory) will not be examined and levels 5, 6, and 7 will be combined into two levels only.

Supplementary Table 4 Antibody List

Antibody	Vendor	Catalog #
RAT ANTI HUMAN CD3	AbD Serotec	MCA1477T
Recombinant Anti-HLA A antibody	abcam	ab52922
Ghost dye BV510	Tonbo Biosciences	13-0870-T100
mCD45 APC-eFluor 780	eBioscience	47-0451-82
hCD45 BV421	BD Biosciences	563879
hCD3 APC	BD Biosciences	557597
hCD4 PerCP/Cy5.5	BioLegend	317428
hCD8 BV785	BioLegend	301046
hCD14 BV786	BD Horizon	563698
hCD16 APC-Cy7	Biolegend	302018
hCD19 BUV737	BD Biosciences	612756
hCD20 PE-Cy5	BioLegend	302308
CD25 BV786	Biolegend	302638
hCD45RA APC	BioLegend	304112
hCD45RO PE	BioLegend	304206
CD56 FITC	BD Pharmingen	562794
hCD62L BV650	BioLegend	304832
hCD69 PE-Cy7	BioLegend	310912
hTSLPR PE-Cy7	BioLegend	322810
hHLA-A2 FITC	BD Biosciences	551285
